# Adrenal Neuroblastoma in an Adult: Effect of Radiotherapy on Local Progression after Surgical Removal

**DOI:** 10.1155/2016/2657632

**Published:** 2016-07-27

**Authors:** Satoshi Kurokawa, Kentaro Mizuno, Akihiro Nakane, Yoshinobu Moritoki, Hidenori Nishio, Hideyuki Kamisawa, Yasue Kubota, Atsushi Okada, Noriyasu Kawai, Yutaro Hayashi, Takahiro Yasui

**Affiliations:** ^1^Department of Nephro-urology, Nagoya City University Graduate School of Medical Sciences, 1 Kawasumi, Mizuho-cho, Mizuho-ku, Nagoya 467-8601, Japan; ^2^Department of Urology, Nagoya Tokushukai General Hospital, 2-52 Kozoji-cho-kita, Kasugai 487-0016, Japan

## Abstract

Here, we report the case of a 62-year-old man with neuroblastoma, which is extremely rare in adults. His tumor was resected, but it recurred four months later. Radiotherapy reduced tumor size, and the patient remained in good health three years after surgical tumor removal. The residual tumor and the treatments administered to this patient were evaluated. We have also reviewed the literature.

## 1. Introduction

Neuroblastoma is one of the most common tumors in children, but it is incredibly rare in adults. The disease in adults differs from that in children with respect to tumor markers, treatment, and prognosis. An adult case of neuroblastoma generally has a poor prognosis, and no standard therapy has been established. In general, adult neuroblastoma is only minimally responsive to current therapeutic schemes.

## 2. Case Presentation

**Figure 1:**
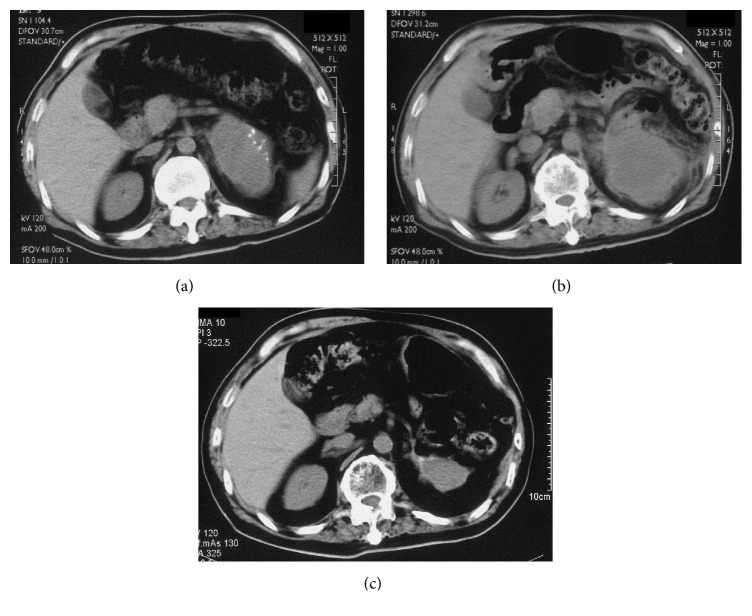
Abdominal computed tomography (CT) image of a left adrenal mass. (a) A preoperative CT scan revealing a 7 × 6 cm tumor with calcification. (b) A CT scan taken 4 months after surgical tumor removal showing a 12 × 9 cm recurrent tumor. (c) Three years after radiotherapy, the residual tumor was 4 × 3 cm.

A left adrenal mass was discovered in a 62-year-old male patient during evaluation for mild epigastralgia that had lasted for several days. He also had a 1-year history of mild hypertension, which was not treated. The patient denied having headaches, palpitations, or excessive/inappropriate perspiration. His physical examination was unremarkable, and no cushingoid features, petechiae, or abdominal striae were detected. Serum electrolytes, blood urea nitrogen, creatinine, and a complete blood count were normal. Pertinent adrenal serum and urine chemistries were also normal, except for a slight elevation in serum norepinephrine. An abdominal X-ray revealed an irregular mass with calcification just above the left kidney. Computed tomography (CT) confirmed the presence of a 7 × 6 cm mass containing sporadic calcification that was located in the left suprarenal position above the left kidney (Figure 1(a)). The mass had infiltrated the pancreas and the spleen. The mass was hetero- and hypointense on T2-weighted magnetic resonance imaging (MRI). ^131^I-metaiodobenzylguanidine (MIBG) scintigraphy did not reveal any MIBG accumulation.

**Figure 2:**
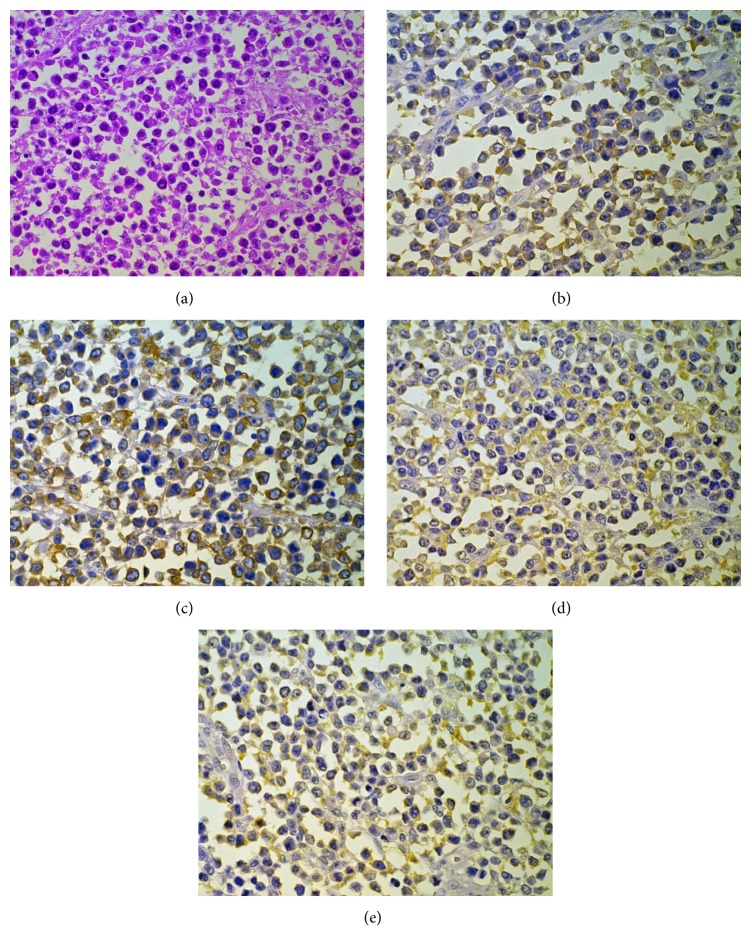
Photomicrographs revealed various tumor properties. (a) The tumor contained small hyperchromatic cells mixed with a finely fibrillar stroma (hematoxylin and eosin). The tumor stains positively for (b) neuron-specific enolase, (c) vimentin, (d) synaptophysin, and (e) S-100 protein. All presented images were reduced from ×400 magnification images.

The patient underwent a left adrenalectomy and a splenectomy that was performed via a thoracoabdominal incision. The mass was partially encapsulated, but the inferior and posterior portions were strongly adherent to adjacent tissues. Gross inspection showed that the tumor measured 8.5 × 5 × 4 cm and 115 g. The cut surface revealed a grayish solid portion and a dark reddish necrotic portion. Histopathologically, the tumor was composed of eosinophilic filamentous tissues and small round cells with hyperchromatic nuclei and scant cytoplasms (Figure 2(a)). Immunohistochemical staining was positive for neuronal/neuroendocrine markers, neuron-specific enolase (NSE, Figure 2(b)), vimentin (Figure 2(c)), synaptophysin (Figure 2(d)), and S-100 protein (Figure 2(e)). However, the tumor was negative for lymphoid markers, the B cell marker UCHL-1, and the T cell marker L26. Histopathological and immunohistochemical examinations confirmed the neuroblastoma diagnosis.

Four months after surgical removal of the tumor, CT revealed a recurrent abdominal mass that was 12 × 9 cm in size (Figure 1(b)). The patient also experienced a concurrent increase in serum NSE levels, which rose to 26 ng/mL (normal range: 0–10 ng/mL). The patient received 50 Gy radiation therapy, after which both the tumor size and serum NSE levels gradually decreased.

Three years after surgery, the patient remains well without any metastases. The mass has decreased to 20% of its original size, with the central necrotic portion of the tumor measuring larger and the peripheral parenchymal portion measuring smaller (Figure 1(c)). Serum NSE levels have been steady, at approximately 6.1 ng/mL. The patient has been followed up using watchful waiting. If the residual mass enlarges again, the patient will undergo an additional surgery to remove the tumor.

## 3. Discussion

Neuroblastoma is a relatively common solid tumor in children, comprising about 8% of all childhood cancers [1]. Over 90% of neuroblastomas are seen in children under the age of 10 years [2]. This could be because the adrenal medulla and sympathetic ganglia are completely differentiated by 10 to 15 years of age. Therefore, neuroblastomas are extremely rare in adolescents and adults.

A literature review revealed that there have been 190 adolescent and adult cases of neuroblastoma described from 1987 to 2014 [3–7], with patients ranging in age from 16 to 77 years. These patients do not include those with intracranial primary tumors, esthesioneuroblastoma, or ganglioneuroma. The distribution of neuroblastomas reflects sympathetic nervous system anatomy in children, adolescents, and adults. In the reviewed 190 patients, the primary tumor site was cephalic and cervical in 93 patients (49%), abdominal in 42 patients (22%), thoracic in 19 patients (10%), pelvic in 16 patients (8%), and in an extremity in 11 patients (6%). An additional 9 patients (5%) had multiple tumors present at the time of diagnosis with no identifiable primary site. Although we cannot be certain, the distribution of primary neuroblastoma sites in adolescents and adults roughly parallels that seen in children.

Neuroblastoma in adolescents and adults has been reported to lack MYCN oncogene amplification, which occurs in 20–30% of younger children. Elevation of catecholamine metabolite secretions, including urinary vanillylmandelic acid (VMA), homovanillic acid (HVA), and dopamine, occurs in as many as 90% of pediatric patients [4] but is generally not seen in adolescents and adults. As in our patient, the serum NSE level is generally used as a tumor marker [5].

The majority of neuroblastomas are irregularly shaped, lobulated, and unencapsulated on CT and MRI imaging. They also sometimes invade adjacent organs or encase adjacent vessels. Neuroblastomas tend to be nonhomogeneous because of tumor necrosis and hemorrhage. They also contain calcifications in approximately 85% of cases, as determined using CT imaging [8].

The rarity of neuroblastomas in adolescents has led to the lack of systematic trials for chemotherapeutic agents, either alone or as a part of combination therapy. CYVADIC (cyclophosphamide, vincristine, adriamycin, and dimethyl triazeno imidazole carboxamide) and James (cyclophosphamide and vincristine) regimens have been administered to adults, but these protocols have not been analyzed because of limited data. Castleberry et al. [9] claimed that radiotherapy was effective in children older than 1 year of age. Moreover, radiotherapy can control local disease in adults with inoperable neoplastic disease and be palliative for painful metastases.

Spontaneous maturation and tumor regression have been documented at a much higher rate in patients with neuroblastoma than those with any other type of tumor [10, 11]. Neuroblastomas in infants can mature into ganglioneuroblastomas or ganglioneuromas and then spontaneously regress. Three hypotheses have been used to explain the spontaneous regression of these tumors, including the immunological theory, the delayed apoptosis theory, and the maturation theory. In our case, the favorable outcome could have resulted from neuronal maturation after radiotherapy. Follow-up CT scans showed that the mass was a gradually shrinking, relatively homogeneous, well-circumscribed oval that was hypoattenuated relative to muscle. The mass also had a cystic feature, which may be explained by an abundance of myxoid matrices that are typical of ganglioneuromas. The residual tumor was not removed, and there was no histopathological evidence of differentiation into a mature ganglioneuroma. However, radiation therapy may have triggered a change in the tumor, based on the observed primitive tumor morphological characteristic reversions in some cases. Therefore, the patient needs to be followed up closely to monitor the presence of primitive tumor structures.
